# Pulsatilla

**Published:** 1888-04

**Authors:** Edward Adams


### PULSATILLA.

Probably no remedy in our *Materia Medica* is more used and abused than this. It has the reputation of being eminently a "woman's medicine," and this not alone among the laity, but, unfortunately, also among many physicians, even some called homœopathic.

This reputation accounts for its too frequent abuse and naturally for the unsatisfactory attendant results. Fortunately for suffering humanity, this drug possesses a number of well-marked or characteristic symptoms in its pathogenesy, so that its unindicated prescription is more inexcusable than in the vast majority of our other remedies.

Thus its mental symptoms first attract our attention and we find it indicated for the *mild, gentle, tearful, yielding, timid,* or *changeable.*

Like the flower itself, affected by every wind, it changes its place continually. So, in the symptoms its use produces, we find a tendency to constant change, not only in degree but also in locality—pains here and then there and nowhere long; quickly changing in degree from the unbearable to the easily borne, so that, like an April day, we have alternations of smiles and showers.

It is necessary also that we bear in mind that for this remedy to be indicated we need not necessarily have the above sensitive, easy-going disposition in the patient so long as the sufferings produce similar phases of character, etc.

In post-partum or other accidental hemorrhages we will be led to think of this remedy, if the patient present the above mentioned characteristics—is full of grief and submissiveness—over-sensitive, low-spirited, and cast down. The flow is changeable like the patient and is either *thick, black, clotted* (easily coagulable), or *thin,* watery, or sometimes the one condition obtains, again the other.

In post-partum secondary hemorrhages, *from retained placenta or coagula,* experience has ofttimes proved its beneficial action.

To complete the picture we must briefly mention the *craving for fresh air, must have the doors and windows open; worse in a warm or close room,* though at the same time is *inclined to be chilly* even in a warm room.

The breathing is difficult. (Puls. is the only remedy in which *difficult breathing accompanies diseased conditions in parts not involved in the act of breathing.*)

Thirst is rare; where it exists we find (as in Arsen. alb.) a craving for small quantities of drink frequently repeated and disturbing the stomach.

EDWARD ADAMS.

